# Antimicrobial Activity of Ethanolic Propolis Extracts from Tame (Arauca) on Oral Biofilm Co-Cultures

**DOI:** 10.3390/pathogens14100982

**Published:** 2025-09-27

**Authors:** Ana Isabel Moreno-Florez, Claudia Maria Bedoya-Correa, Claudia Garcia, Alejandro Pelaez-Vargas

**Affiliations:** 1 Faculty of Dentistry, Universidad Cooperativa de Colombia, Medellín 055422, Colombia; 2 Universidad Nacional de Colombia, Medellín 050034, Colombia

**Keywords:** *Apis mellifera*, *C. albicans*, Co-cultures, *S. mutans*, *S. sanguinis*, propolis

## Abstract

Oral diseases such as dental caries, stomatitis, and periodontitis are closely associated with biofilms that are resistant to conventional therapeutic approaches. *Streptococcus sanguinis* and *Streptococcus mutans* play a key role as primary and secondary colonizers of oral surfaces, respectively, and interact synergistically with other species, including *Candida albicans*, to promote the establishment and progression of infection. Objective: To evaluate the antimicrobial activity of ethanolic extracts of propolis from Tame (Arauca) on biofilms formed in co-cultures from reference strains and co-cultures with clinical isolates of oral pathogens. Methodology: Propolis was collected from *Apis mellifera* hives placed in rural Tame (Arauca), located in the foothills of the Eastern Andes (Colombia). Ethanolic extracts of propolis (EEP) were prepared in a 0.07 g/mL concentration and biological characterization was performed on single and complex co-cultures of *S. mutans* (*serotype c*), *S. sanguinis*, and *C. albicans* using disc diffusion test, determination of MIC and BMC, growth curves and biofilm formation. The cell viability and metabolic activity of primary cell cultures derived from a dental pulp explant were evaluated using the MTT assay. Results: EEP exhibited higher inhibition zones than chlorhexidine against *S. mutans* and *C. albicans* and lower efficacy against *S. sanguinis*. Among the microorganisms evaluated, *S. mutans* showed the lowest MIC and BCM values, followed by *C. albicans* and *S. sanguinis*. Growth curves and biofilm formation assays revealed higher inhibition in co-cultures of reference strains (*S. mutans* + *C. albicans*), while multi-species cultures (*S. mutans* + *S. sanguinis* + *C. albicans*), or clinical strains (*S. mutans clinical isolated* + *S. sanguinis* + *C. albicans*), showed higher resistance. Cell viability assays revealed low cytotoxicity (<30%) in primary cell cultures. Conclusions: EEPs exhibited antimicrobial activity against relevant oral pathogens, especially in simple co-cultures, supporting their potential as natural therapeutic alternatives. However, their efficacy decreases in the presence of clinical strains and complex co-cultures, highlighting the importance of considering these variables in the development of oral treatments.

## 1. Introduction

Oral diseases represent a substantial global public health challenge. These diseases include dental caries, gingivitis, periodontitis, stomatitis and peri-implantitis, and they are commonly associated with the formation of oral microbial biofilms. According to the World Health Organization (WHO), dental caries is the fourth most expensive chronic disease to treat worldwide, exhibiting a substantial impact on public health, individual economy, and reduction in quality of life [1].

The oral microbiota plays an essential role in maintaining microbial diversity, as well as an essential role in maintaining homeostasis of the oral ecosystem. The microbiomes that comprise this ecosystem are organized into structured biofilms embedded in a matrix of extracellular polymers derivate from salivary proteins and microorganisms, which adhere to soft and hard oral surfaces [2]. In the absence of pathologic influences, commensal species prevail within the intestinal tract, exerting a synergistic effect with the host’s immune system to reduce the proliferation of pathogens. However, intricate interactions among the microbiota, the oral microenvironment, and the host response might disrupt this equilibrium, leading to a state of dysbiosis [3,4].

Dental biofilms are composed of a microbial consortia that colonize tooth surfaces embedded in an extracellular matrix of polysaccharides and proteins [5]. These bacterial communities actively modify the oral niche, generating an acidic microenvironment that favors the dominance of cariogenic species, such as *Streptococcus mutans* and *Lactobacillus* spp. [6]. The chronic acidic environment created by biofilms might produce enamel demineralization, with subsequent development of caries lesions (white spots to cavities) in advanced stages [7,8]. On the other hand, periodontitis is characterized by an imbalance that results in an increase in Gram-negative anaerobic species. These species have been demonstrated to induce a chronic inflammatory response, which contributes to connective tissue attachment loss and bone resorption [9,10,11].

*S. mutans* is a Gram-positive and facultative anaerobic bacterium that is associated in approximately 30% of the microbiota in patients with dental caries [12]. This species has demonstrated the capacity to survive and reproduce in acidic environments generated by sugar fermentation [13,14,15]. Furthermore, it has been observed to colonize environments that are becoming more acidic, suggesting its adaptability to changing environmental conditions [16,17]. *S. mutans* demonstrate high genotypic and phenotypic variability, which facilitates the expression of various virulence factors associated with biofilm formation, acidogenesis, and aciduricity. These characteristics are pivotal in its role in the development of dental caries [12]. *S. mutans* is classified into four serotypes (c, e, f, and k), based on the presence of rhamnose-glucose antigenic polysaccharides anchored to the cell wall [17]. Studies conducted on clinical isolates show the wide distribution of these serotypes across the oral cavity.

*Serotype c* is the most prevalent, followed by serotypes e and f, while serotype k is the least frequent [16,18,19,20,21]. In general, *S. mutans* strains are distinguished by their high acidogenic capacity, as explained above. However, clinical isolates belonging to *serotype c* are more acidogenic than other serotypes, which significantly contributes to their virulence. *S. mutans* might interact synergistically with other species, such as *Candida albicans*. Their interactions reduce pH and modulate other oral pathologies, such as stomatitis [22,23]. Similarly, their association increases the grade of maturation, thickness, and virulence of dental biofilms [24].

*C. albicans* establishes synergistic interactions with other oral streptococcal species that enhance its invasive capacity [15]. *S. sanguinis* is an early colonizer of the oral cavity that plays a fundamental role in coaggregation with secondary colonizers, promoting the formation of heterotypic biofilms, which contribute to the development of complex microbial communities on dental surfaces [25]. However, *S. mutans* exhibits antagonistic characteristics against *S. sanguinis*, thus showing competition for initial colonization of the tooth surface [25].

Currently, in vitro monoculture models for studying oral biofilms have advanced from reference strains to clinical isolates, which retain their resistance mechanisms and might be representative of the oral micro-environment [26,27]. In comparison to reference strains, clinical isolates of *S. mutans* also exhibited considerable phenotypic variability, thereby validating their utilization in multispecies co-culture models to replicate more complex interactions in vitro [28,29,30].

The challenge of effectively managing dental plaque is still an open field, and even though mechanical methods (e.g., toothbrushing, flossing and professional cleaning) are widely regarded as the backbone of oral biofilm management, their effectiveness might be compromised by patient-related factors, such as adherence to treatment and reduced fine motor skills, and limited access to dental services [31,32]. In order to reduce these limitations, synthetic antibiotics, with varying degrees of efficacy, have been utilized as chemical adjuncts for the management of biofilms. However, the indiscriminate use of antibiotics has contributed to the emergence of resistant strains through bacterial mutations and horizontal gene transfer [33,34,35,36,37]. In this context, natural products have gained interest as anti-biofilm agents with components such as polyphenols, flavonoids, and terpenes emerging as promising candidates due to their broad-spectrum antimicrobial activity and ability to target biofilm virulence mechanisms against several microbial species [38,39,40].

Among natural products, propolis is recognized for its antimicrobial and anti-inflammatory properties. This resinous substance, collected by bees to protect their hives, has been incorporated into oral care formulations due to its antibacterial, antifungal, and low-toxicity properties [41,42,43,44,45]. However, the biological activity of propolis varies according to its geographical and botanical origin [46,47], presenting diverse mechanisms of action that affect multiple microbial structures, thereby allowing its efficacy against a wide range of bacteria, including antibiotic-resistant strains [48,49,50]. In this context, the antimicrobial properties of propolis have generated growing interest in the dental field. A substantial number of studies have demonstrated its efficacy against bacterial and fungal oral pathogens [51,52,53,54,55,56], thereby supporting its potential as a therapeutic agent in the management of oral infections. In vitro assays in this field have been focused on the analysis of the inhibitory effect of propolis on individual strains and planktonic cultures. As such, several studies have demonstrated that cells in the biofilm state exhibit significantly greater resistance to antimicrobial agents compared to their planktonic counterparts [57].

The objective of this study was to evaluate the antimicrobial activity of the ethanolic extracts of propolis from Tame (Arauca) on biofilms formed in co-cultures from reference strains and co-cultures with clinical isolates of oral pathogens.

## 2. Materials and Methods

Propolis was collected by scraping from *Apis mellifera* hives in the rural area of Tame (Arauca), a remote municipality located in the eastern Orinoquía region of Colombia. This area lies on a plateau formed by the foothills of the Eastern Andes and is characterized by limited infrastructure, low population density, and restricted accessibility, offering a naturally preserved environment. The hives belonged to the local beekeeper’s association (Colmiel, Tame, Colombia). Samples were stored at 4 °C before characterization tests.

- Ethanolic Extract of Propolis (EEP)

Samples of 10 g of raw propolis were submitted for alcoholic extraction using 100 mL of absolute ethanol (Merck, Darmstadt, Germany). They were immersed in an ultrasound bath for 1 h, followed by incubation at room temperature for 24 h. Filtration was performed using 25-µm Whatman filter paper (Cytiva, Marlborough, MA, USA). The solvent was evaporated in a reduced-pressure roto evaporator (Heidolph, Schwabach, Germany) at 40 °C to one-third of the initial volume.

To quantify the final concentration of propolis, 10 mL of EEP was dried at 180 °C using a thermal moisture balance (JM Science, Beijing, China). The stable weight value was defined when the variation in the weight was lower than 0.01 mg for 10 min. The final EEP had a 0.07 g/mL concentration and was stored in dark conditions at 4 °C, following protocols to maintain extract stability and bioactivity [58,59].

- Microbial strains and growth conditions

Reference strains of *Streptococcus mutans* (*serotype c*, ATCC 25175, USA), *Candida albicans* (ATCC 90028), and *Streptococcus sanguinis* (ATCC 10556) were used. In addition, a clinical isolate of *S. mutans serotype c* (P20S4), obtained from a saliva sample of a patient with caries, was characterized and identified by molecular testing [16]. These strains were reactivated in Brain Heart Infusion Agar (BHI Agar, Scharlau, Barcelona, Spain) and incubated at 37 °C in microaerophilia for 48 h with 5% CO_2_. At the end of the incubation time, microbial suspensions were prepared by transferring microbial cells into 10 mL of BHI broth (Brain Heart Infusion, Scharlau, Barcelona, Spain), until a value of 90 ± 5 Nephelometric Turbidity Units (NTU) was obtained using a turbidimeter (TB1, Velp Scientific, Usmate Velate, Italy). This value is equivalent to a concentration of approximately 1.5 × 10^8^ CFU/mL (Colony Forming Units per milliliter). Standard cell concentrations were obtained as recommended by the Clinical and Laboratory Standard Institute (CLSI) [60]. Subsequently, the cell suspensions were diluted 1:100 in 0.9% saline solution to obtain a final concentration of 1.5 × 10^6^ CFU/mL.

- Antimicrobial Activity of ethanolic extracts of propolis

Single co-cultures were prepared with a mixture of two strains: reference strains *S. mutans* and *C. albicans* (*Sm*+*Ca*), clinical isolate of *S. mutans* P20S4 (*Sm_Ci_*) and *C. albicans* (*Ca*), *S. sanguinis and C. albicans* (*Ss*+*Ca*). In addition, complex co-cultures were prepared using a combination of three strains: reference strains *S. mutans*, *C. albicans* and *S. sanguinis* (*Sm*+*Ca*+*Ss*), clinical isolate of *S. mutans* P20S4, *C. albicans* and *S. sanguinis* (*Sm_Ci_*+*Ss*+*Ca*). Co-cultures were performed at the same ratio with a cell concentration of 1 × 10^6^ CFU/mL.

To determine the susceptibility of the evaluated strains against different concentrations of EEP, an agar diffusion test was performed using the Kirby-Bauer method [61]. Co-culture cell suspensions were inoculated on Mueller-Hinton agar plates (MH, Scharlau, Barcelona, Spain). Three filter paper discs of 6 mm diameter (Advantec, Osaka, Japan) impregnated with 10 μL of the EEPs were placed on the agar, 0.2% chlorhexidine digluconate (CHX, Farpag, Bogotá, Colombia) was used as a positive control, and 0.9% saline was used as a negative control. In addition, discs impregnated with absolute ethanol (EtOH) were seeded to evaluate the inhibitory effect of the solvent present in the extracts. *S. mutans* and *S. sanguinis* plates were incubated at 37 °C in microaerophilia (5% CO_2_) for 24 h, while those of *C. albicans* were incubated under aerobic conditions. The diameter of the growth inhibition zone (including the 6 mm disk) was measured in millimeters using the calibrated measurement tool in Fiji (version 2.14.0), the open-source distribution of ImageJ software [62].

- Minimum Inhibitory Concentration (MIC) and Minimum Bacteriostatic Concentration (MBC)

For the minimum inhibitory concentration (MIC) and minimum bacteriostatic concentration (MBC) assays, an inoculum of each species was prepared up to a concentration of 1 × 10^6^ CFU/mL, as described above, and serial dilutions of 1:10 were performed until the desired concentration was obtained. Also, 96-well polystyrene plates (Costar, Corning Inc., New York, NY, USA) were used, and 100 μL of the ethanolic extracts of propolis (EEP) was added in the first 2 rows of the plates and then 100 μL of the inoculum. Serial dilutions of the extract were made from 80 to 0.004 mg/mL (1:1 *v*/*v*) and added following the same proportions, dispensed from the most concentrated to the least concentrated solution. CHX (0.2%) was used as a positive control, and BHI broth inoculated with cell suspensions without addition of EEP was used as a negative control. The plates were incubated for 24 h at 37 °C in an atmosphere containing 5% CO_2_ [60,63]. After 24 h, the viability of microorganisms was evaluated using a tetrazolium salt reduction method, 3-(4,5-dimethylthiazol-2-yl)-2,5-diphenyltetrazolium bromide (MTT, Life technologies, Carlsbad, CA, USA) [36,38]. The MIC was defined as the lowest EPP concentration at which no growth was detected. The minimum bactericidal concentration (MBC) was obtained by seeding 20 μL of the dilution (corresponding to the 1X, 2X and 4X MIC) on BHI agar plates in triplicate. MBC was selected as the lowest EPP concentration at which the substance prevents noticeable growth of microorganisms (greater than 3 CFU/mL). The evaluations were performed in triplicate at three separate times. The results obtained from the EPP were compared with controls.

- Growth curves in the presence of extract

Bacterial growth curves were obtained in 96-well polystyrene plates using co-cultures at a concentration of 1 × 10^6^ CFU/mL prepared using the previously described methodology [60], 50 µL of the EEPs were added to each well, and a volume of 200 µL was obtained using the inoculated medium. In addition, BHI broth was seeded with titrated EEP solutions for absorbance blank subtraction, and inoculated BHI was seeded with the co-cultures as a positive control for microbial growth. Some wells were seeded with 50 µL of CX to contrast the effect of the EEPs. Absorbance measurements were performed every 30 min for 18 h at an optical density (OD) of 600 nm using a microplate spectrophotometer (Multiskan GO, Thermo Scientific, Waltham, MA, USA). The data were used to construct growth curves and to compare the effect of EEP versus biofilm formation in the co-culture conditions.

- Reduction in biofilm formation

To evaluate the reduction in biofilm formation of the co-cultures in the presence of the EEPs, 24-well polystyrene plates (Costar, Corning Inc., New York, NY, USA) with 100 µL of EEP and 900 µL of each of the prepared seeded co-cultures were used. CHX and saline solution were used as positive (C+) and negative (C−) controls, respectively. The plates were incubated for 24 and 48 h at 37 °C in a 5% CO_2_ atmosphere.

After the incubation time had elapsed, the culture medium was carefully removed and three washes with saline were performed to remove unattached cells. Then, 500 µL of 0.1% crystal violet solution was added, and the cells were incubated for 15 min at room temperature. Three washes with saline solution were performed to remove excess dye and 500 µL of 95% ethanol was added. Subsequently, the sample was left in gentle agitation for 15 min, and the absorbance was read at OD 570 nm in a microplate spectrophotometer. The results are expressed as percentages of biofilm reduction. All assays were performed in triplicate.

- Cytotoxicity assay

The MTT assay (Life Technologies, Carlsbad, CA, USA) was used to evaluate the cytotoxicity of EEPs. The test was performed using a primary cell culture obtained from a dental pulp explant obtained from an orthodontic patient undergoing mandatory exodontia. Dental pulp from the extracted tooth was obtained by maceration and trypsinization processes to achieve cell isolation. Cell culture was allowed to progress to 80% confluence before trypsinization. Cells were cultured in Dulbecco’s modified Eagle’s medium (DMEM, Bio&Sell, Feucht, Germany) with 2. 3% (*v*/*v*) stable glutamine, 10% (*v*/*v*) fetal bovine serum (Bio&Sell, Feucht, Germany), and 1% (*v*/*v*) penicillin/streptomycin. The cultures were incubated at 37 °C in a 5% CO_2_ atmosphere.

To evaluate the cytotoxicity of the EEPs, 100 µL of the EEPs was seeded in a 96-well plate and 100 µL of the cell culture was added at a density of 2500 cell/cm^2^. In addition, control wells were included, where 100 µL of the culture medium and 100 µL of the cell culture were seeded. In addition, wells were seeded with 100 µL of pure DMSO (Merck, Darmstadt, Germany) and 100 µL of the cell culture, which were used as negative controls. Cytotoxicity was evaluated after 24 and 48 h of culture incubation using an MTT culture medium ratio (1:10). Subsequently, cells were incubated for an additional 4 h, and MTT was discarded. DMSO was added, and the absorbance was measured at OD 570 nm. Experiments were performed in triplicate. The results are expressed as percentage viability.

- Statistical Analysis

The results were evaluated using the one-factor ANOVA method of analysis of variance, with a comparison of means with the Tukey 95% confidence DHS statistic. In all cases, *p* < 0.05 values were considered indicative of statistically significant differences. Analyses were performed using IBM SPSS Statistics (version 28, IBM Corp., New York, NY, USA).

## 3. Results

The inhibition zones of the extracts against each species of interest are shown in Table 1. The EEPs exhibited antimicrobial effect against all the evaluated strains, and their effect was significantly greater than that of CHX against *S. mutans* strains. In the case of *S. sanguinis*, the EEPs showed an inhibitory effect, but it was significantly lower than that of the positive control. Against *C. albicans*, the EEPs had a similar effect to that of the positive control. The ethanol used as a solvent in the extracts had a very slight inhibitory effect against all the evaluated strains, being significantly lower than the effect of the EEP and the positive control. The saline solution (C-) used as a negative control showed no activity against the evaluated strains.

Upon calculation of the MIC and BMC of EEP, significantly higher values were obtained against *S. sanguinis* than against the other strains. MIC values of 2.1 ± 0.05 mg/mL against *S. mutans* strains, 2.5 ± 0.08 mg/mL against the clinical isolate of *S. mutans*, 3.8 ± 0.03 mg/mL against *C. albicans* and 7.9 ± 0.09 mg/mL against *S. sanguinis* were obtained. The BMC assay results obtained were 4.2 ± 0.03 mg/mL against the *S. mutans* strain, 5.0 ± 0.03 mg/mL against the clinical isolated *S. mutans*, 7.6 ± 0.05 mg/mL against *C. albicans* and 15.8 ± 0.02 mg/mL against *S. sanguinis*.

**Figure 1:**
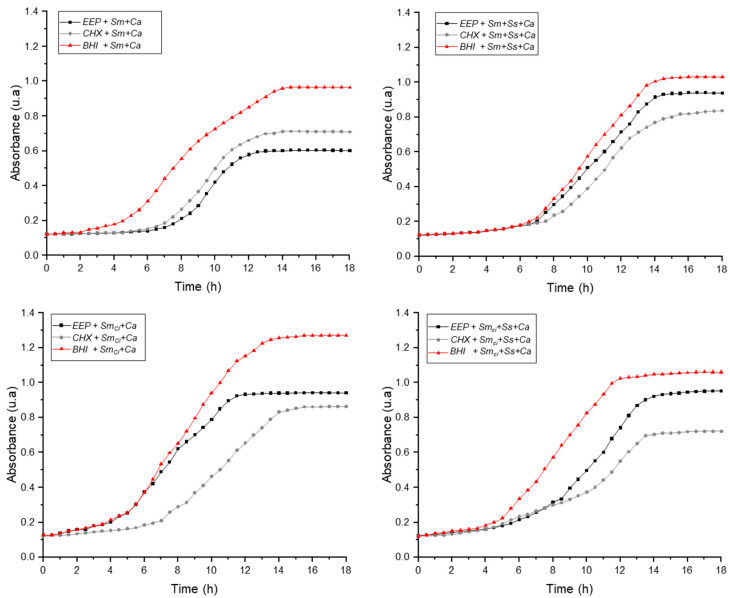
Co-culture growth curves in presence of Propolis Extracts (EEP), Chlorhexidine (CHX) and co-cultures without antimicrobial agents. Single co-cultures: reference strains *S. mutans* and *C. albicans* (*Sm*+*Ca*), clinical isolate of *S. mutans* P20S4 and *C. albicans* (*Sm_ci_*+*Ca*). Complex co-cultures: reference strains *S. mutans, S. sanguinis* and *C. albicans* (*Sm*+*SS*+*Ca*), and clinical isolate of *S. mutans* P20S4, *S. sanguinis* and *C. albicans* (*Sm_ci_*+*Ss*+*Ca*).

**Figure 2:**
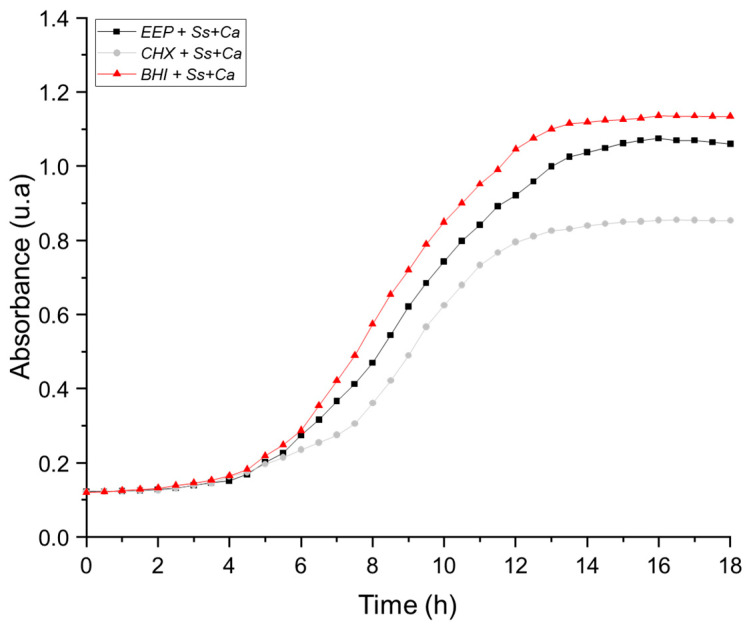
Growth curves in presence of Ethanolic Extracts of Propolis (EEP), Chlorhexidine (CHX) and without *treatment*. Single co-culture of *S. sanguinis* and *C. albicans* (*Ss*+*Ca*). A partial reduction in growth was observed with EEP, which was less pronounced than that obtained using CHX.

The growth curves of co-cultures in the presence and absence of ethanolic extracts of propolis (EEP), CHX (C+), and without antimicrobial agents are presented in Figure 1 and Figure 2. Figure 1 shows the behavior of the cultures after 18 h of incubation. In the co-culture formed by reference strains *S. mutans* and *C. albicans* (*Sm*+*Ca*), a noticeable growth inhibition was evidenced in the presence of EEP, reaching absorbance values even lower than those observed with CHX, especially during the exponential phase. This result indicates the remarkable antimicrobial effect of the natural extract against this microbial combination. On the other hand, when the reference strain of *S. mutans* was replaced by the clinical isolate of *S. mutans* P20S4 (*Sm_ci_*), the inhibitory effect of EEPs was reduced compared with CHX. However, a reduction in growth relative to the untreated control was maintained, demonstrating that EEPs retain a significant degree of activity against clinical strains, although with lower relative efficacy.

The complex co-cultures (*Sm*+*SS*+*Ca* and *Sm_ci_*+*Ss*+*Ca*) showed lower overall sensitivity to EEPs. Although a reduction in growth was observed during the first hours of culture (lag phase and beginning of the exponential phase), by the end of the log phase, absorbance levels approached those of untreated controls.

Figure 2 shows the curves obtained for single co-cultures with *S. sanguinis* and *C. albicans* (*Ss*+*Ca*) evaluated in the presence of EEP, CHX and without antimicrobial agents. The results show that *S. sanguinis* presents a lower sensitivity to EEP than the other evaluated microorganisms. Although a partial reduction in growth was observed during the exponential phase and toward the end of the culture, this decrease was significantly lower than that induced by CHX, indicating the limited efficacy of the extract against this specific co-culture. In contrast, CHX treatment produced a marked and sustained growth inhibition throughout the incubation period, confirming its high antimicrobial potency against the combination of *sanguinis* and *C. albicans* (*Ss*+*Ca*).

**Figure 3:**
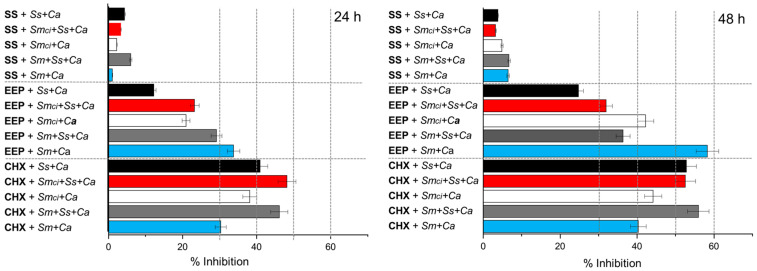
Percentage of inhibition in biofilm formation. Single co-cultures: reference strains *S. mutans* and *C. albicans* (*Sm*+*Ca*), clinical isolate of *S. mutans P20S4* and *C. albicans* (*Sm_ci_*+*Ca*), and *S. sanguinis* and *C. albicans* (*Ss*+*Ca*). Complex co-cultures: reference strains *S. mutans*, *S. sanguinis* and *C. albicans* (*Sm*+*Ss*+*Ca*); clinical isolate of *S. mutans* P20S4, *S. sanguinis* and *C. albicans* (*Sm_ci_*+*Ss*+*Ca*), after 24 and 48 h of incubation. Cultures were treated with Ethanolic Extracts of Propolis (EEP), Chlorhexidine (CHX, C+), or saline solution (SS, C−). The results express the percentage of biofilm inhibition with respect to the untreated control.

Figure 3 shows the percentage of biofilm inhibition observed in the different combinations of co-cultures after 24 h and 48 h of incubation with EEP, CHX (C+), and saline solution (C−). At 24 h, the complex co-cultures *Sm*+*SS*+*Ca*, treated with EEP and CHX, exhibited the highest inhibition levels, exceeding 25%, with CHX showing the most pronounced effect. At 48 h, *Sm*+*SS*+*Ca* treated with EEP and CHX exhibited even greater inhibition, exceeding 35% and 55%, respectively. This trend suggests a time-dependent enhancement of antimicrobial activity.

In single co-cultures, *Sm*+*Ca* and *Sm_ci_*+*Ca* treatment with EEP also produced significant inhibition, exceeding 40% and reaching values up to 55% in the case of *Sm*+*Ca* exposed to EEP at 48 h. This last combination represented the condition with the greatest inhibitory effect among all evaluated treatments.

These results show a general trend toward increased inhibition in the presence of EEP, particularly after 48 h of exposure. Likewise, variability was observed with the type of co-culture, with *Sm*+*Ca* and *Sm_ci_*+*Ca* co-cultures being the most sensitive to EEP treatment. However, it is relevant to highlight that in the co-cultures, including the clinical isolate of *S. mutans* (*Sm_ci_*, P20S4), the inhibitory effect of EEP was considerably lower when compared to the results shown by the co-cultures of the reference *S. mutans*, showing greater resistance to the effect of EEP. Finally, co-cultures involving *S. sanguinis* exhibited a trend toward lower inhibition values.

**Figure 4:**
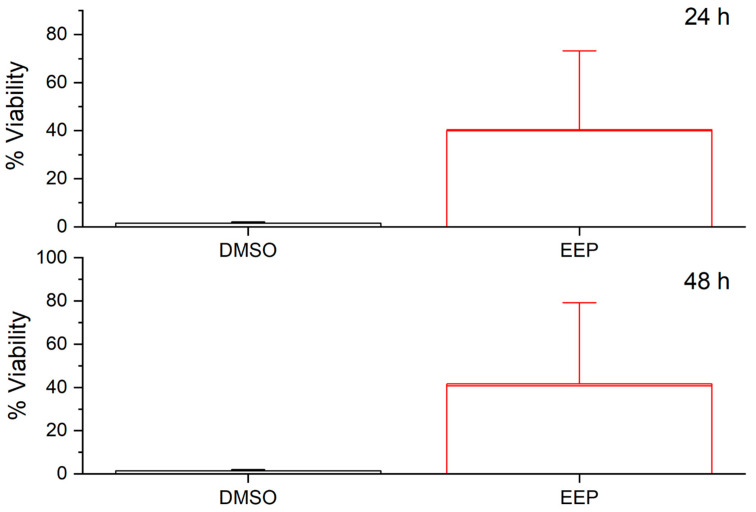
Percentage of cell viability determined by MTT assay in primary cell cultures exposed to ethanolic extracts of propolis (EEP) and DMSO (C-) for 24 and 48 h. The results show high viability in EEP-treated cells (>70%) in both incubation periods, whereas exposure to DMSO resulted in a viability of less than 7%, indicating its cytotoxic effect.

Figure 4 shows the results of the analysis of the cytotoxic effect on a primary cell culture derived from a dental explant after exposure to pure DMSO and ethanolic extracts of propolis (EEP). It was observed that cell viability in the presence of EEP was greater than 70% at both 24 and 48 h of incubation, indicating the low cytotoxicity of this treatment under the evaluated conditions. In contrast, cells exposed to pure DMSO had a viability lower than 7%, indicating a marked cytotoxic effect.

## 4. Discussion

The objective of this work was to test the antimicrobial effectiveness of Ethanolic Extracts of Propolis (EEP) from the region of Tame (Colombia) against co-cultures of reference strains *S. mutans*, *S. sanguinis* and *C. albicans*, including a clinical isolate of *S. mutans* from a patient with active dental caries. EEPs exhibited antimicrobial activity against all co-cultures tested although different levels of sensitivity were observed. Specifically, co-cultures including *S. sanguinis* were less sensitive to the action of EEPs, which might be related to the natural ability of this species to compete in oral environments via competitive exclusion mechanisms.

The obtained results agree with previous studies reporting an inhibitory effect of propolis against the bacterial and fungal strains used in this study [64,65,66,67,68,69]. This antimicrobial activity is associated with the presence of phenolic compounds (flavonoids, esters, and terpenes) [43,47,70,71,72]. In particular, flavonoids present in propolis from this region [68] have the ability to inhibit the activity of the enzyme glucosyltransferase, which is directly involved in the processes of *S. mutans* adherence to the tooth surface, which is a key step in the formation of dental plaque. In addition, these compounds have demonstrated antifungal activity against *C. albicans*, thereby reinforcing their therapeutic potential in oral mixed infections [73,74,75,76]. However, the mechanisms of action of propolis are not limited to enzyme inhibition. Multiple effects on the cell structure and function of microorganisms have been reported, such as disintegration of the cytoplasm, rupture of membranes and cell walls, partial bacteriolysis, loss of membrane potential due to increased permeability, and inhibition of protein synthesis [74,76,77,78]. This variety of mechanisms could explain the efficacy observed against mixed cultures, where microbial interactions can modify the overall susceptibility of the system.

The assessment of the antimicrobial effect in multispecies cultures is particularly relevant because it reproduces the microecosystems present in the oral cavity more realistically. It has been reported that *S. mutans* infection levels increase significantly in the presence of *C. albicans* due to a synergistic interaction that facilitates biofilm formation and maturation [22]. On the other hand, there are also antagonistic interactions, such as those observed between *S. mutans* and *S. sanguinis*, which compete for the early colonization of tooth surfaces. This competition may be influenced by the order of colonization (i.e., if *S. mutans* is the first to attach); it may establish an ecological advantage over *S. sanguinis* [25].

The differences observed between the reference strain of *S. mutans* and the clinical isolate might be highlighted. The latter exhibited greater resistance to the action of EEP, suggesting that genetic factors, adaptation to the oral environment, and previous exposure to antimicrobial compounds may modify the susceptibility of the strains [79,80]. This finding underscores the importance of including clinical strains in antimicrobial susceptibility studies because they more closely represent real infectious conditions.

This study was conducted using a simplified in vitro model based on a limited number of microbial species, which represents only a partial approximation to the complexity of the oral ecosystem. Although oral diseases involve multispecies microbial communities and are influenced by factors such as genetic susceptibility, host immune response, and environmental conditions, as explained above, the experimental design employed allowed for a preliminary evaluation of the antimicrobial effect of propolis extract on selected oral pathogens under co-culture conditions. These limitations should be taken into account when interpreting the results and underscore the need for future research using more complex models that incorporate multiple microbial species, as well as environmental and host-related variables, to simulate conditions that are more closely aligned with the clinical field. Furthermore, these findings reinforce the potential of propolis as a natural antimicrobial agent, particularly in the context of complex oral infections. However, it is necessary to continue evaluating the standardization of its chemical composition, as well as its effectiveness on in vivo and clinical models, to consolidate its therapeutic application.

## 5. Conclusions

The results of this study demonstrated that Ethanolic Extracts of Propolis (EEP) from the Tame region (Colombia) exhibited remarkable antimicrobial activity against co-cultures of *S. mutans*, *S. sanguinis* and *C. albicans*, including clinically relevant strains in the context of dental caries.

The efficacy of EEPs varied depending on the composition of the co-culture, being less effective against combinations including *S. sanguinis*, suggesting a modulatory role of microbial interactions in susceptibility to natural agents.

## Figures and Tables

**Table 1:** Inhibition Zones (IZ) of propolis extracts (EEP), chlorhexidine (CHX), saline (C-) and solvent (ET) against *S. mutans*, *S. mutans* isolate (P20S4), *C. albicans*, and *S. sanguinis* strains.

		IZ (mm)		
Samples	*S. mutans*	*S. mutans* _clinical isolated_	*C. albicans*	*S. sanguinis*
EEP	31.75 ± 2 ^A a^	32.40 ± 0.1 ^A a^	31.02 ± 4 ^A a^	9.76 ± 0.3 ^A b^
CX	17.64 ± 1 ^B^	20.45 ± 0.06 ^B^	29.63 ± 5 ^A^	14.60 ± 3 ^B^
EtOH	7.21 ± 2 ^C^	8.11 ± 0.01 ^C^	10.06 ± 1 ^B^	6.57 ± 1 ^C^
C-	0 ± 0 ^D^	0 ± 0 ^D^	0 ± 0 ^C^	0 ± 0 ^D^

^A–D^ Measurements with the same uppercase letter within a column indicate no statistically significant differences (*p* < 0.05). ^a–b^ Measures with the same lowercase letter within a row indicate no statistically significant differences (*p* < 0.05).

## Data Availability

The data that support the findings of this study are available from the corresponding author upon reasonable request.
